# Research on Train-Induced Vibration of High-Speed Railway Station with Different Structural Forms

**DOI:** 10.3390/ma17174387

**Published:** 2024-09-05

**Authors:** Xiangrong Guo, Jianghao Liu, Ruibo Cui

**Affiliations:** School of Civil Engineering, Central South University, Changsha 410083, China; ljhcsuorst@163.com (J.L.); crbcsu@163.com (R.C.)

**Keywords:** integrated station-bridge structure, combined station-bridge structure, train-induced vibration, dynamic response

## Abstract

Elevated stations are integral components of urban rail transit systems, significantly impacting passengers’ travel experience and the operational efficiency of the transportation system. However, current elevated station designs often do not sufficiently consider the structural dynamic response under various operating conditions. This oversight can limit the operational efficiency of the stations and pose potential safety hazards. Addressing this issue, this study establishes a vehicle-bridge-station spatial coupling vibration simulation model utilizing the self-developed software GSAP V1.0, focusing on integrated station-bridge and combined station-bridge elevated station designs. The simulation results are meticulously compared with field data to ensure the fidelity of the model. Analyzing the dynamic response of the station in relation to train parameters reveals significant insights. Notably, under similar travel conditions, integrated stations exhibit lower vertical acceleration in the rail-bearing layer compared to combined stations, while the vertical acceleration patterns at the platform and hall layers demonstrate contrasting behaviors. At lower speeds, the vertical acceleration at the station concourse level is comparable for both station types, yet integrated stations exhibit notably higher platform-level acceleration. Conversely, under high-speed conditions, integrated stations show increased vertical acceleration at the platform and hall levels compared to combined stations, particularly under unloaded double-line working conditions, indicating a superior dynamic performance of combined stations in complex operational scenarios. However, challenges such as increased station height due to bridge box girder maintenance, track layer waterproofing, and track girder support maintenance exist for combined stations, warranting comprehensive evaluation for station selection. Further analysis of integrated station-bridge structures reveals that adjustments in the floor slab thickness at the rail-bearing and platform levels significantly reduce dynamic responses, whereas increasing the rail beam height notably diminishes displacement responses. Conversely, alterations in the waiting hall floor slab thickness and frame column cross-sections exhibit a minimal impact on the station dynamics. Overall, optimizing structural dimensions can effectively mitigate dynamic responses, offering valuable insights for station design and operation.

## 1. Introduction

In recent years, with the acceleration of urbanization, the demand for public transport in many places between central cities and their peripheral new towns has been increasing, and the existing transportation can no longer meet the travel needs of the public. Therefore, there is an urgent need for a transportation mode with a large capacity, fast speed, and smooth operation to solve the new public transportation demand in cities. As an effective means to alleviate the pressure on urban roads and meet the traffic demand between cities, the elevated rail transit scheme has the advantages of less road-traffic interference, a high passenger capacity, a low construction cost, and a short construction cycle [1]. As an important part of the elevated line, the elevated station not only plays the role of allowing people to access transportation, but also has a greater commercial value, which can make up for its operation and maintenance costs.

Feldmann et al. [2,3] established a computational model of the Berlin train station and investigated the vibration response of the bridge and roof structure when the train passes through. Zhu et al. [4,5,6] analyzed the random vibration characteristics of each floor of the same station by vehicle action based on the dynamics model of the train-rail-station, and explored the law of the station’s random vibration change with the vehicle speed. Li and Ran et al. [7,8] focused on the Chongqing Shapingba Comprehensive Transportation Hub, and employed frequency domain loading analysis to investigate the vibration response patterns of the platform and station building. Xie et al. [9] through measurements of the station building’s structural vibrations and noise responses during train arrivals and departures, analyzed the response patterns of the “integrated bridge and building” type of subway side-platform elevated station. Ba et al. [10] through on-site measurements, conducted a comparative analysis of structural vibrations caused by different train speeds, vibrations at different measurement points, and vibrations generated during the passage of two trains. This study provides a basis for the simulation and prediction of high-speed railway stations with the “integrated station-bridge” structural form. Minsu et al. [10,11,12] employed finite element analysis to study the vibration attenuation patterns of elevated railway stations. Cai et al. [13] through on-site measurements, analyzed the vibration responses of elevated stations during trains’ arrival, departure, and passage. Xia et al. [14] investigated the mechanism of elevated structure vibrations under train loading, analyzing the impact of vibrations from different types of elevated bridges on the environment. Ju et al. [15] through on-site measurements, studied the ground vibration characteristics induced by trains traveling at different speeds on elevated railways. Yang et al. [16] analyzed the impact of the train speed, distance from the vibration source, and train type on the vibration of structural floors. Tanabe et al. [17] proposed an effective computational method for analyzing the dynamic interaction between high-speed trains and railway structures. Meng et al. [18] focused on Shangrao station, using a finite element model validated with real data. The results showed that vertical vibrations were stronger than horizontal ones. Parallel train traffic caused more vibrations than grade-separated traffic. Higher speeds and heavier trains increased vibrations. These insights provided valuable support for improving future station designs. Cui et al. [19] constructed a coupled system of elevated stations and track structures, obtaining the vibration response of elevated stations and the mechanical performance of track structures under dynamic loading. Zhao et al. [20] starting from the structural characteristics of stations with different forms, combined with the functions and requirements of station buildings and equipment, proposed supplementary and improved suggestions for current specifications. Ma et al. [21] focused on train-induced vibrations at Beijing Fengtai Railway Station, Asia’s largest double-story high-speed station. A vibration source load model and a finite-element model of the station were established and verified using field data. The study analyzed the station’s vibration under different track positions and train speeds in both time and frequency domains. Deng et al. [22] conducted an analysis of the displacement, magnitude, and distribution patterns of a station platform under typical train-induced conditions.

The current literature is rich in studies on the vibrational behavior of monolithic station-bridge systems under various dynamic loads. However, there is a notable lack of research on the dynamic characteristics of semi-separated station-bridge systems, which feature a separation between the track beam and station platform. This gap in the research is significant, as these systems may have unique dynamics not addressed by studies on integrated structures. Addressing this gap is crucial for enhancing our understanding of the vibrational comfort and structural integrity of elevated stations, which are essential to modern transportation networks. Our research aims to provide a comparative analysis of the dynamic responses of different elevated station designs, contributing to the optimization of station structures for efficiency, safety, and passenger comfort.

## 2. Project Background of Station-Bridge

At present, the structural forms of elevated stations are mainly divided into separated station-bridge structures, combined station-bridge structures, and integrated station-bridge structures. Compared with the first option, the latter two options have wider engineering application prospects as new elevated station structures. A track girder simply supported on the station structure is called a station-bridge combination structure; while the track frame and the bearing layer of the elevated station both forming a whole frame structure is called a station-bridge unity structure. The track and the bearing layer of the station forming a whole frame is called an integrated station-bridge structure, while the track beams being simply supported on the station is called a combined station-bridge structure. An elevated station is a special structural system that combines a building and a bridge, and it has no specific code for design verification. Therefore, a large number of scholars have conducted a series of studies on this structure. Table 1 describes the advantages and disadvantages of the two-bridge building-integrated stations.

**Figure 1:**
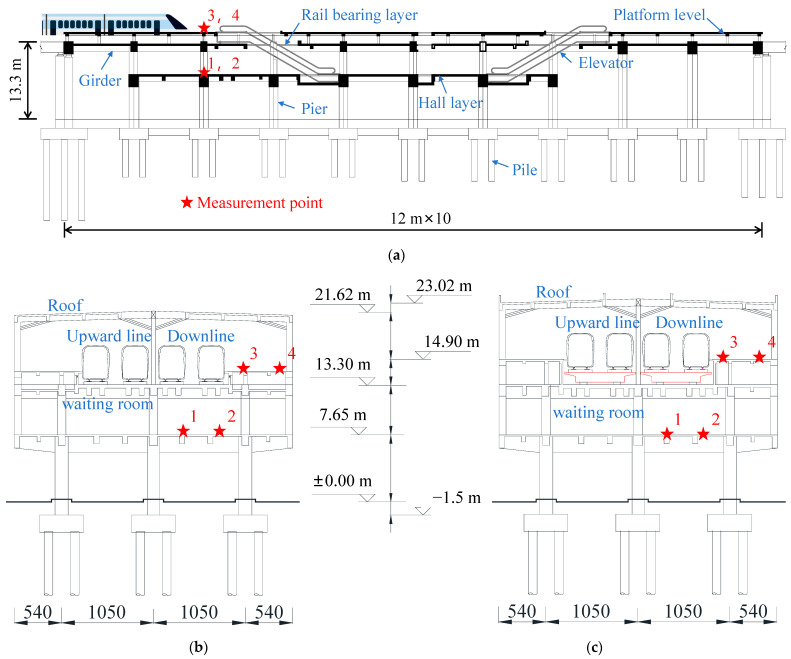
Schematic diagram of station-bridge structure. (**a**) Schematic elevation of station-bridge structure (hiding roof structure); (**b**) section of integrated station-bridge structure; (**c**) section of combined station-bridge structure.

An elevated station on an urban rail transit line in China is taken as the background of this study. Two construction options were considered at the design stage of this elevated station, the first one was an integrated station-bridge structure and the second one was a combined station-bridge structure. The first option was finally adopted considering the construction period as well as the building cost. The longitudinal schematic diagram of the elevated station structure is depicted in Figure 1a. The cross-sectional schematic diagrams of the integrated station-bridge structure and the combined station-bridge structure are illustrated in Figure 1b,c.

The station is designed for a speed of 120 km/h, accommodating a four-track passenger-dedicated line. The passing method employed is the middle passing approach. The station building adopts a fully cast-in-place spatial frame structure system. The main structure of the station consists of three levels, arranged from bottom to top as the concourse level, track-bearing level, and platform level. The roof structure is constructed using concrete. The heights for the respective levels of the combined station-bridge structure, measured from the ground upwards, are 7.65 m, 13.30 m, and 16.30 m. The total height falls within the range of 23–24 m. The floor heights of the integrated station-bridge structure, measured from the ground upwards, are 7.65 m, 13.30 m, and 14.90 m for the respective levels. The total height falls within the range of 21–22 m. The station has a total length of 120 m and a width of 31.80 m. Three rows of columns are arranged laterally, with a spacing of 10.5 m between each pair of columns. The columns have an overhang of 5.4 m on their outer sides. The platform width on each side is 7.8 m. The longitudinal column network is arranged in 10 spans of 12 m without deformation joints. The end beams of the station share piers and columns with the bridge structure, both supported on simple supports by the bridge piers.

## 3. Analytical Model of Train-Track-Station System

### 3.1. High-Speed Train and Track Irregularity

**Figure 2:**
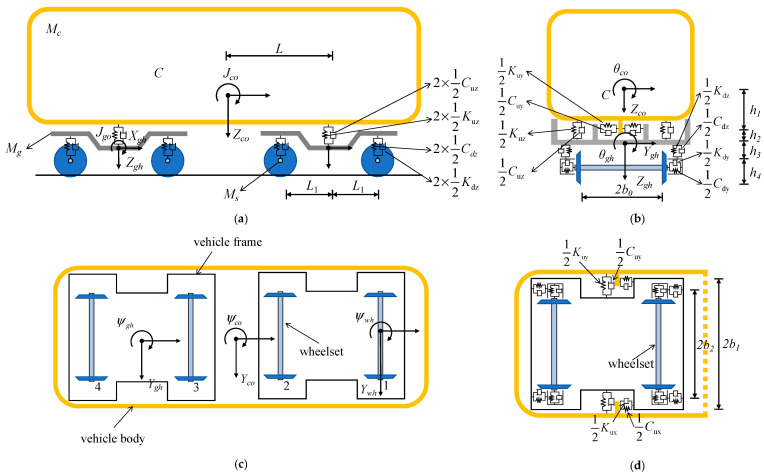
The topology model of the high-speed train: (**a**) elevation view; (**b**) side view; (**c**) plan view; (**d**) schematic diagram of the vehicle’s horizontal spring.

A railroad vehicle is a multi-degree-of-freedom system consisting of components such as the body, bogies, wheelsets, and suspension. In the theory of vehicle multibody dynamics, the stiffness of the components, such as the vehicle body, the bogie, and the wheels, far exceeds the stiffness of the suspensions in the first and second systems. Therefore, the elastic deformation of the components can be ignored, and the lateral and longitudinal acceleration of the vehicle body can be analyzed more effectively. The following assumptions are used in the analysis of the vehicle space vibration analysis model:

(1) The body, bogie, and wheelset are assumed to be rigid;
(2) The longitudinal vibration of locomotives and vehicles, and their effects on bridge vibration and travel speed, are not considered;
(3) Vibrations in the wheelsets, bogies, and body are relatively minor;
(4) The train suspension system is simulated with linear springs and viscous damping;
(5) The wheelset and steel rail are in close contact in the vertical direction, and the vertical displacement of the wheelset and the rail are identical;

The topology model of a high-speed vehicle is depicted in Figure 2. The spatial vibrations of the vehicle body and frame include five degrees of freedom (DOF): lateral swing, floating, rolling, pitching, and yawing. Forth-and-back motion is not considered. Each wheelset has two DOF: lateral swing and yawing. Therefore, each four-axle vehicle has a total of 23 degrees of freedom.

The relative position of the train to the bridge is always changing as the train travels over the bridge. Based on the two basic theories above, it is possible to derive the stiffness, mass, damping matrix ([*K_v_*], [*M_v_*], [*C_v_*]), and load vector quantity {*P_v_*} of the train. Based on the set-in-right-position rule of matrix formation, the overall stiffness matrix [*K*], mass matrix [*M*], damping matrix [*C*], and load array {*P*} of the train-bridge system at any moment t can be calculated by combining the train and bridge dynamic matrices.

These can be deduced from D’Alembert’s principle for the total potential energy of the *j*th carriage.

(1) $$\Pi_{vj}=V_{mj}+V_{Fj}+U_{Ej}+U_{Ewj}+V_{gj}$$

The total vibration potential energy of the *j* train on the bridge at *t* time can be expressed as:

(2) $$\prod_{v}=\sum_{j=1}^{N}\prod_{vj}=\sum_{j=1}^{N}[V_{msj}+V_{mgj}+V_{mcj}+V_{Fu1j}+V_{Fu2j}+V_{Fu3j}+V_{Fd1j}+V_{Fd2j}+V_{Fd3j}+V_{Fdj}+U_{Eu1j}+U_{Eu2j}+U_{Eu3j}+U_{Ed1j}+U_{Ed2j}+U_{Ed3j}+U_{E\omega j}+V_{gj}]$$

where $V_{mj}$ is the work of inertial forces on the car’s body, bogie, and wheelset. $V_{Fj}$ is the work of the damping suspension systems, $U_{Ej}$ is the energy of deformation at the damping suspension systems. $U_{ej}$ is the strain energy at the damping suspension systems. $U_{Ewj}$ is the gravitational potential energy at the wheelsets. $V_{gj}$ is the gravitational potential energy at the car body.

By calculating the total potential energy of a vehicle and applying the principle of the standing value of potential energy and the law of “seating” which forms the matrix, the equations of operation of the vehicle are calculated as follows:

(3) $$\left[M_{v}\right]\left\{{\ddot{\delta }}_{v}\right\}+\left[C_{v}\right]\left\{{\dot{\delta }}_{v}\right\}+\left[K_{v}\right]\left\{\delta_{v}\right\}=\left\{P_{v}\right\}$$

In this paper, the train is modeled as a prototype of China’s B-type metro vehicle, and the relevant parameters of the vehicle are shown in Table 2 below.

Production errors in the rail, wear due to long-term train operation, uneven elasticity of the rail, sleepers, and ballast, as well as variations in the foundation, can lead to changes in the geometric configuration of the track. These factors result in differences between the actual geometric shape of the track in the field and its ideal state. Track irregularities can be classified into four types: vertical profile irregularity, alignment irregularity, cross-level irregularity, and gauge irregularity. The wavelength characteristics of track irregularities are described and rated using power spectral density. In this study, the American track irregularity spectrum is used to simulate the internal excitation between the train and the railway station. The spectrum is obtained by actually measuring the data of the U.S. track in different states and then fitting the mathematical formula as follows:

Vertical profile:
  (4) $$S_{v}\left(\Omega \right)=\frac{kA_{v}\Omega_{c}^{2}}{\Omega^{2}\left(\Omega^{2}+\Omega_{c}^{2}\right)}$$
Alignment:
  (5) $$S_{a}\left(\Omega \right)=\frac{kA_{a}\Omega_{c}^{2}}{\Omega^{2}\left(\Omega^{2}+\Omega_{c}^{2}\right)}$$
Cross-level and gauge:
  (6) $$S_{c}\left(\Omega \right)=S_{g}\left(\Omega \right)=\frac{4kA_{v}\Omega_{c}^{2}}{\left(\Omega^{2}+\Omega_{c}^{2}\right)\left(\Omega^{2}+\Omega_{s}^{2}\right)}$$
  where $S_{c}\left(\Omega \right)$ and $S_{g}\left(\Omega \right)$ are, respectively, the PSD functions of the cross-level and gauge track irregularities [cm^2^/(rad/m)], and $\Omega_{s}$ is the cutoff frequency (rad/m). The standard track spectra are divided into six grades, and parameters of grade-6 are listed in Table 3. In the table, the maximum allowable train speeds related to different grades in accordance with the running safety standard are also given.

### 3.2. Finite Element Models of Track-Station System

**Figure 3:**
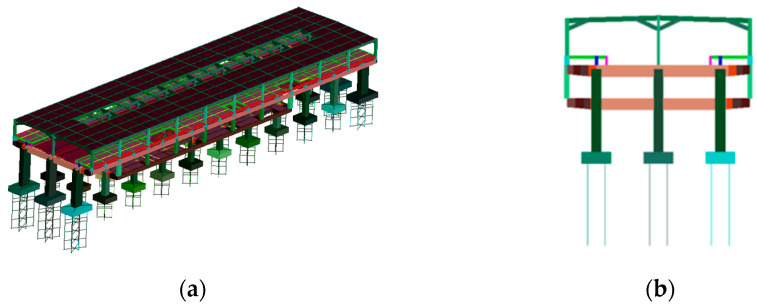
Finite element model of integrated station-bridge system: (**a**) axonometric view; (**b**) front elevation.

**Figure 4:**
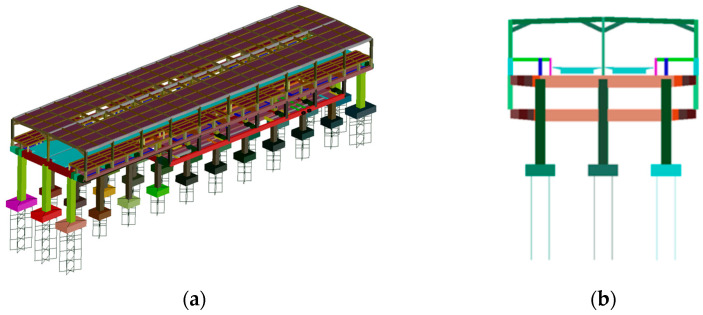
Finite element model of separated station-bridge system: (**a**) axonometric view; (**b**) front elevation.

This study utilized a finite element model of the track-station structure established using bridge dynamics analysis software developed by Central South University. The structural components of the station building, including the floor slabs simulated using shell elements and other structures simulated using beam elements, were modeled. All materials were assumed to be linearly elastic, with the elastic modulus E for concrete grades C35, C40, and C45 taken as 3.15 × 10^4^ N/mm^2^, 3.25 × 10^4^ N/mm^2^, and 3.35 × 10^4^ N/mm^2^, respectively. For all concrete types, Poisson’s ratio was assumed to be 0.2. The concrete materials used in the model include C35 concrete for the platform-level floor slabs and pile foundations, C40 concrete for platform-level concrete beams, columns, hall-level concrete beams, floor slabs, canopy columns, beams, and diagonal braces, and C45 concrete for track-level beams, columns, and slabs. Raleigh damping with a damping ratio of 2% was employed to simulate the damping of the station structure. In the foundation structure modeling, the piles were considered as elastic foundation beams, with the soil resistance around the piles being simulated by calculating the stiffness of soil springs. Figure 3 and Figure 4 show the finite element model of station-bridge system.

The frame beams of the station structure are modeled using spatial beam elements, where each beam element has two nodes, and each node possesses three translational displacement degrees of freedom and three rotational degrees of freedom. On the other hand, the floor slabs of the station structure are modeled using shell elements, with each shell element having four nodes, and each node having two degrees of freedom. The total number of nodes, beams, and shell elements for each model is shown in the Table 4.

**Figure 5:**
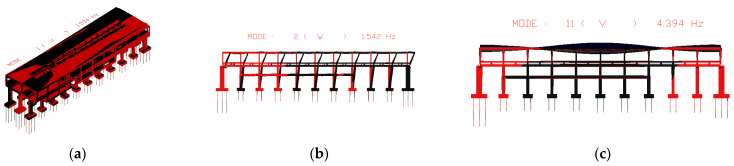
First-order vibration pattern of integrated station-bridge system. (**a**) first mode; (**b**) second Mode; (**c**) eleventh Mode.

**Figure 6:**
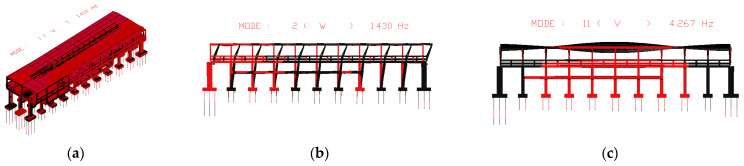
First-order vibration pattern of separated station-bridge system. (**a**) First mode; (**b**) second mode; (**c**) eleventh mode.

Using the track-station finite element model established earlier, the first 100 orders of structural vibration patterns were obtained through modal analysis. Among the two different structures, the first orders of the horizontal bending, vertical bending, and longitudinal drift vibration mode are the same, while the size of the self-oscillation frequency is slightly different. The specific values are shown in Table 2, and two different types of stations are plotted in the first order of the vibration mode diagram shown in Figure 5 and Figure 6. The first 11 vibration modes of these two types of stations are shown in Table 5.

### 3.3. Train-Track-Station Coupling Model

In this paper, the train-rail-station (hereinafter referred to as the car-rail station) system is regarded as a coupled time-varying integral system, the static equilibrium position of each monolithic structure is taken as the coordinate origin, the boundary conditions of the station structure are taken as the boundary conditions of the system, and the complex contact between the wheels and rails is turned into the internal contact of the system, which can exclude the uncertainty and ensure that the equations have a unique solution.

Considering the interrelationship between the spatial vibration displacement of each vehicle and station, the total potential energy of the vehicle-rail station system at any moment *t* is calculated, including the total potential energy of the vehicle $\Pi_{v}\left(t\right)$ and the total potential energy of the track-station $\Pi_{d}\left(t\right)$, and then, using the principle of the total potential energy of the dynamics of the elastic system, the principle of invariant value, the principle of total potential energy with stationary value in elastic system dynamics, and the “set-in-right-position” rule, forming the structural method, the vibration equations of the vehicle-rail station system can be set up at moment *t*:

(7) $$\left[M]\{\ddot{\delta }\}+[C]\{\dot{\delta }\}+[K]\{\delta \}=\{P\right\}$$

where [M], [C], and [K] are the mass, damping, and stiffness matrices of the train-rail station system at time *t*, respectively; $\left\{\ddot{\delta }\right\}$, $\left\{\dot{\delta }\right\}$, and $\left\{\delta \right\}$ are the acceleration, velocity, and displacement columns of the train-rail station system at time *t*, respectively; $\left\{P\right\}$ is the array of loads consisting of the wheel–rail contact force due to the track surface irregularity and the vehicle’s own weight applied to the coupled system at moment *t*.

**Figure 7:**
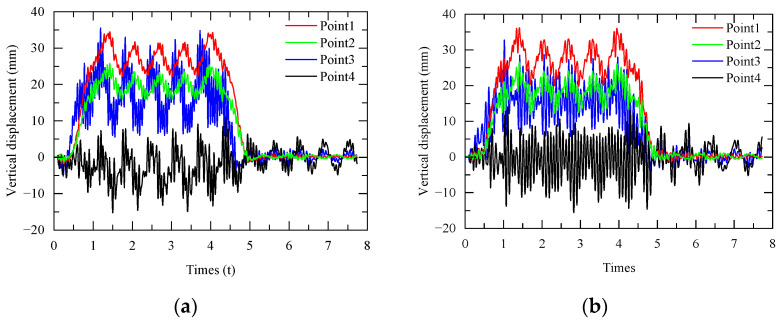
Vertical displacement of station-bridge structures: (**a**) integrated station-bridge structure; (**b**) combined station-bridge structure.

The displacement response of the two station structures under the action of the train is calculated by using the train-station coupling model, established above, as shown in Figure 7 below. It can be found that the vertical displacement curves at the key points of the two station structures are basically the same.

**Figure 8:**
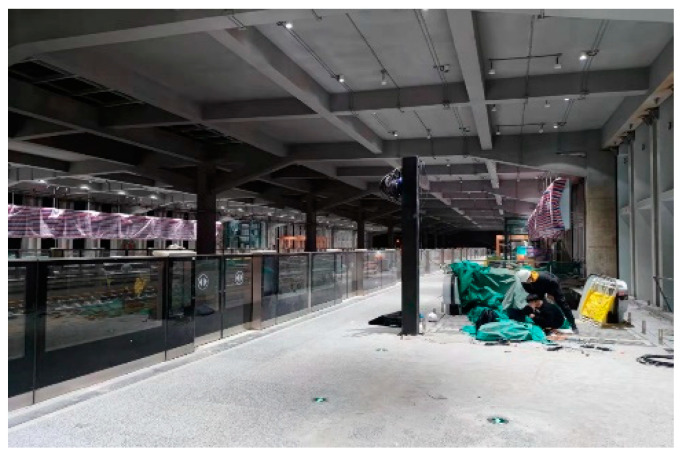
Field test photos of the integrated station-bridge structure.

**Figure 9:**
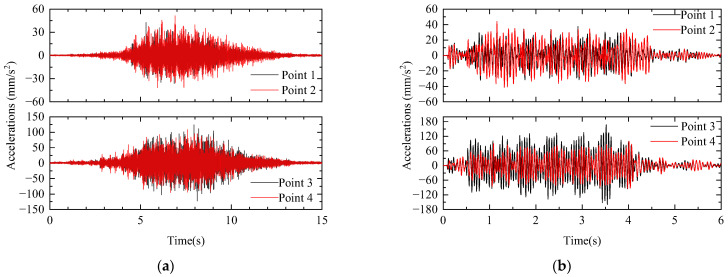
The field test data and theoretical calculation data of integrated station-bridge structure. (**a**) measured acceleration; (**b**) calculated acceleration.

In order to verify the accuracy of the coupling model of the rail station, this paper uses the station-bridge structure constructed by the integrated station-bridge structure program to carry out the real test analysis, and the field test photos are shown in Figure 8. Calculation conditions close to the test conditions are selected to compare the dynamic response of the station structure. In this section, the calculation condition is selected as a train with six train sets passing through the station unloaded at 110 km/h, and the vertical acceleration time series of the floor slabs at the platform level and the station-hall level are compared. The field test data and theoretical calculation data are shown in Figure 9.

A large number of studies have shown that the vibration response of the station structure is mainly controlled by the vertical dynamic load of the train, and the vibration of the station is dominated by the vertical vibration of the vertical acceleration peaks of the measurement points, which are summarized in Table 6. The calculation of this paper’s model and the measured acceleration peaks are relatively close to each other, thus verifying that the spatially coupled vibration-calculation results in this paper have a certain degree of reliability.

## 4. Dynamic Response Analysis for Train-Track-Station System

### 4.1. Relationship between Vehicle Parameters and Station Dynamic Response

Under the effect of train motion loads, the traveling speed, car weight, number of train sets, and train double-line operation will have a large impact on the vibration performance of the station system. According to the above calculation model and calculation principle, the spatial dynamic response of the train-station system of the rail transit B-type vehicle passing through the station under unloaded and fully loaded conditions, respectively, is calculated for the two station analysis models, and the corresponding train groupings and calculation conditions are as follows, as shown in Table 7:

#### 4.1.1. Single-Line Train Operation

**Figure 10:**
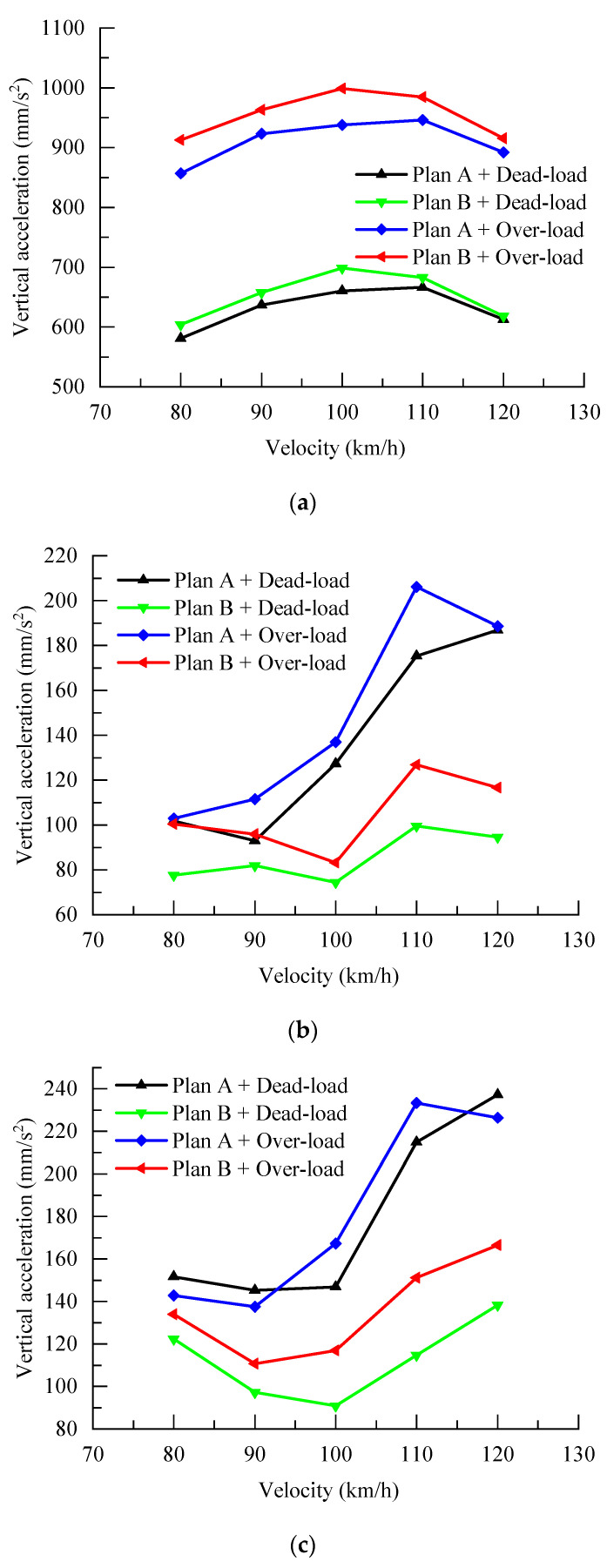
Maximum vertical acceleration of the station under single-line condition. (**a**) Rail-bearing layer; (**b**) hall layer; (**c**) platform level.

Based on the train-station coupled dynamics model established above, the train parameters of the coupled model under the two station schemes are analyzed. In order to more comprehensively evaluate the response of each position of the station structure affected by the train parameters, the peak dynamic responses of the slabs at the rail, platform, and concourse levels, shown in Figure 1, are extracted and analyzed. The vertical accelerations of the slabs at the rail, platform, and concourse levels when the train passes through the station under different speed conditions are shown in Table 8. In order to more intuitively see the effect of the responses of the floor slabs of the track layer, platform layer, and concourse layer by the train speed, the curve of each response index is shown in Figure 10.

From Figure 10, it can be seen that, when a single-track type-B train with a four-car formation, either empty or fully loaded, passes through the station at speeds of 80 km/h, 90 km/h, 100 km/h, 110 km/h, and 120 km/h, the vertical acceleration of the track-supporting layer in the unified station-bridge structure is consistently lower than that of the station-bridge combined structure. Conversely, the vertical acceleration of the hall level in the unified station-bridge structure is consistently higher than that of the station-bridge combined structure. Similarly, the vertical acceleration of the platform level in the unified station-bridge structure is also consistently higher than that of the station-bridge combined structure.

Specifically, when the single-track type-B train with a four-car formation, either empty or fully loaded, passes through the station at speeds of 80 km/h and 90 km/h, the vertical acceleration of the hall level in both types of stations is similar. However, the vertical acceleration of the platform level in the unified station-bridge structure under the single-track empty condition is higher than that of the station-bridge combined structure under both the single-track empty and fully loaded conditions.

At speeds of 100 km/h, 110 km/h, and 120 km/h, when the single-track type-B train passes through the station, the vertical acceleration of both the platform level and the hall level in the unified station-bridge structure under the single-track empty condition is higher than that of the station-bridge combined structure under both the single-track empty and fully loaded conditions. This indicates that the station-bridge combined structure exhibits better dynamic performance at the platform and hall levels under single-track conditions.

#### 4.1.2. Double-Line Train Operation

**Figure 11:**
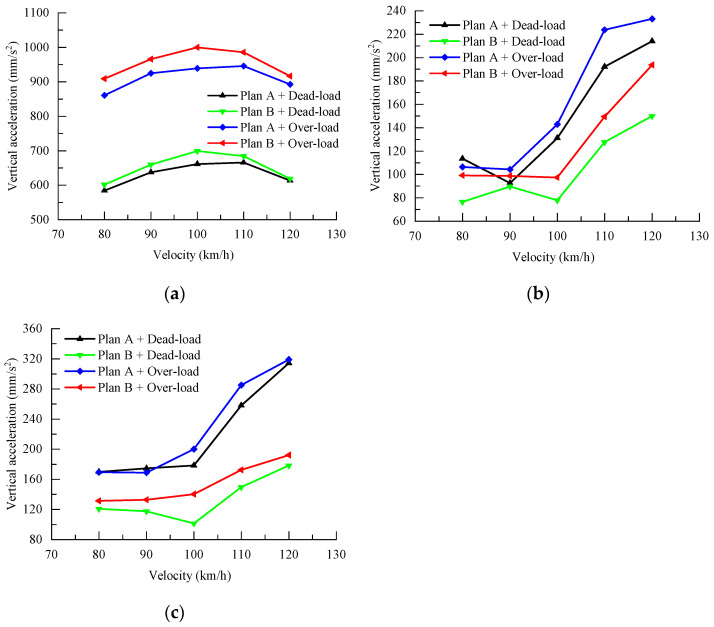
Maximum vertical acceleration of the station under double-line condition. (**a**) Rail-bearing layer; (**b**) hall layer; (**c**) platform level.

The above model is used to calculate the dynamic response of the station structure when an unloaded and fully loaded train passes through the station at a speed of 80~120 km/h under a two-lane condition. The vertical accelerations of the floor slabs of the track layer, concourse layer, and platform layer when the train passes through the station under different speed conditions are shown in Table 9. In order to visualize the responses of the floor slabs at the track level, platform level, and concourse level by the train speed, the curve of each response index is shown in Figure 11.

As can be seen from Figure 11, when the four-unloaded and four-full-loaded double-line B trains pass through the station at the speeds of 80 km/h, 90 km/h, 100 km/h, 110 km/h, and 120 km/h, the vertical acceleration of the bearing layer of the station-bridge unification structure station is smaller than that of the bearing layer of the station-bridge combination structure station, and that of the station-hall layer of the station-bridge unification structure station is larger than that of the station platform layer of the station-bridge combination structure station. The vertical acceleration at the station concourse level of the station-bridge combination structure is larger than that at the station concourse level of the station-bridge combination structure, and the vertical acceleration at the platform level of the station-bridge combination structure is also larger than that at the platform level of the station-bridge combination structure. Among them, when four-unloaded and four-full-loaded B-type trains pass through the station at low speeds of 80 km/h and 90 km/h, the vertical accelerations at the platform level of the two types of stations are similar, while the vertical acceleration at the concourse level of the combined station-bridge structure is obviously larger than that of the combined station-bridge structure. When passing through the station at high speeds of 100km/h, 110 km/h, and 120 km/h, the vertical acceleration at the platform level and the concourse level of the station-bridge unity type of station under no-load double-line operation is obviously greater than that at the platform level and the concourse level of the station-bridge combination type of station under no-load and full-load double-line operation, which also proves that the combined structure has better dynamic performance at the platform level and the concourse level of the station-bridge combination structure in the case of double lines. This also proves that the station-bridge combination structure still has better dynamic performance at the platform level and concourse level in the case of two-lane operation.

### 4.2. Analysis of Station Structural Parameters

In summary, the comprehensive dynamic performance of the structure of Scheme B is better than that of Scheme A. However, Scheme B has the problem that the height of the whole station is increased due to the bridge box girder, and it also has the problems of waterproofing of the track layer and inconvenient maintenance and replacement of the track girder bearings. In order to reduce the dynamic response of the station structure of Scheme A, this chapter investigates the dynamic response of the station structure when the train passes through the station with different heights of track girders, different floor thicknesses, and different cross-sectional sizes of frame columns based on the “bridge-construction” fully integrated station structure, so as to provide a reference for the calculation of the vibration response of more and more station structures of this kind in the future. Taking the working condition of a single-line full-load train passing through the station at 120 km/h as an example, the effects of different member-size parameters on the dynamic response of the station of Scheme A are calculated separately.

#### 4.2.1. Track Girder Height

In this section, five groups of track girder heights of 1.0 m, 1.2 m, 1.4 m, 1.6 m, and 1.8 m are selected to investigate the influence of track-girder height change on the dynamic response of the station structure. Taking the working condition of a single-line full-load train passing through the station at 120 km/h as an example, the maximum values of the dynamic response of the floor slab at the station hall level and that of the floor slab at the station platform level obtained from the calculations are summarized in the following table under the condition of different track girder heights.

**Figure 12:**
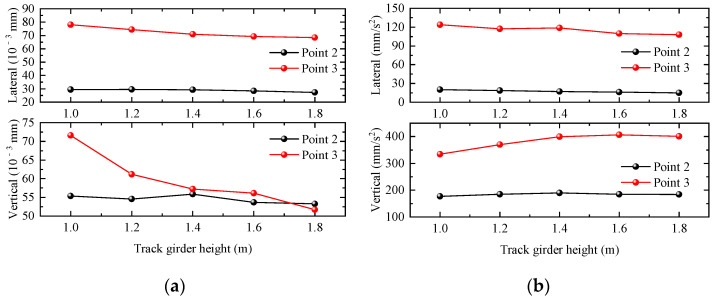
Relationship curve between station dynamic response and track girder height. (**a**) Displacement; (**b**) acceleration.

The maximum values of the dynamic responses of different areas of the station structure are shown in Figure 12. The displacement response of the station concourse floor and platform floor both change greatly with the increase in the track beam height, and the acceleration response is less affected by the track beam height. When the track beam height increases, the vertical displacement response of the station structure has an obvious tendency to decrease, the vertical acceleration of the station-hall level has a tendency to increase, and the vertical acceleration of the waiting hall basically remains unchanged. And the lateral displacement and acceleration responses show a decreasing trend with the increase in the track beam height.

#### 4.2.2. Thickness of Rail-Bearing Layer

**Figure 13:**
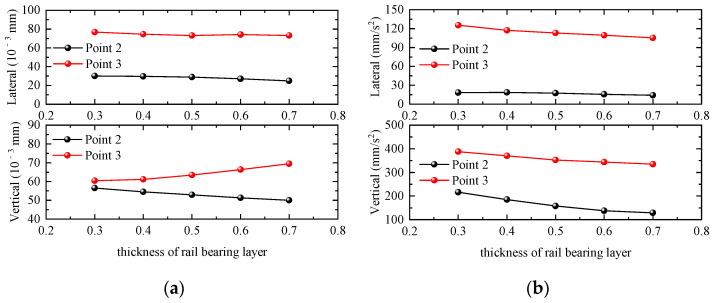
Relationship curve between station dynamic response and thickness of rail-bearing layer. (**a**) Displacement; (**b**) acceleration.

In this section, five groups of track-layer floor-slab thicknesses of 0.3 m, 0.4 m, 0.5 m, 0.6 m, and 0.7 m are selected to investigate the influence of changes in the thickness of the track-layer floor slab on the dynamic response of the station building structure. Taking the working condition of a single-line full-load train passing through the station at 120 km/h as an example, the maximum values of the dynamic response of the waiting-hall floor slab and the platform floor slab are calculated as shown in Figure 13. The displacement responses of the waiting-hall floor and platform floor of the station are small with the increase in the thickness of the floor slab of the track layer, and the acceleration response is greatly affected by the thickness of the floor slab of the track layer. When the thickness of the track layer floor increases, the vertical acceleration of the station structure decreases the fastest, the vertical displacement of the waiting hall decreases with the increase in the thickness of the track layer floor, and the vertical displacement of the platform layer increases with the increase in the thickness of the track layer floor. The transverse acceleration of the platform floor slab decreases slightly with the increase in the thickness of the bearing rail floor slab, and the transverse displacement remains basically unchanged. The transverse displacement and acceleration of the bearing rail floor slab remain basically unchanged with the increase in the thickness of the bearing rail floor slab.

#### 4.2.3. Thickness of Hall Layer

**Figure 14:**
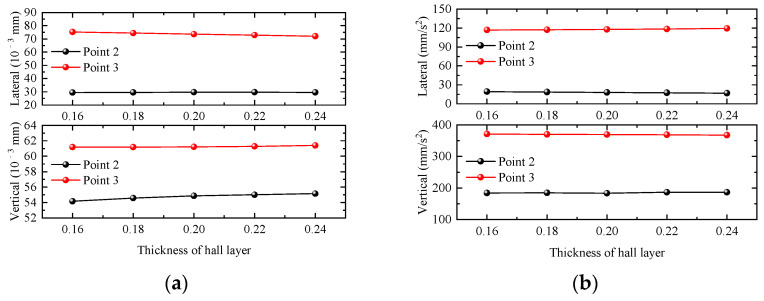
Relationship curve between station dynamic response and thickness of hall layer. (**a**) Displacement; (**b**) acceleration.

In this section, five groups of waiting-hall floor thicknesses of 0.16 m, 0.18 m, 0.2 m, 0.22 m, and 0.24 m are selected to investigate the influence of waiting-hall floor thickness changes on the dynamic response of the station structure. Taking the working condition of a single-line full-load train passing through the station at 120 km/h as an example, the dynamic responses of the waiting-hall floor slab and platform floor slabs calculated under different waiting-hall floor slab thicknesses are shown in Figure 14. The displacement and acceleration responses of the waiting-hall floor slab and platform floor slab of the station are basically unchanged with the increase in the thickness of the waiting-hall floor slab. When the thickness of the waiting-hall slab increases, the velocity and acceleration response of the station waiting-hall and platform slab change very little, and only the vertical displacement of the waiting-hall slab increases slightly, so the responses of the waiting-hall and platform slab are affected very little by the change in the thickness of the waiting-hall slab.

#### 4.2.4. Thickness of Platform Level

**Figure 15:**
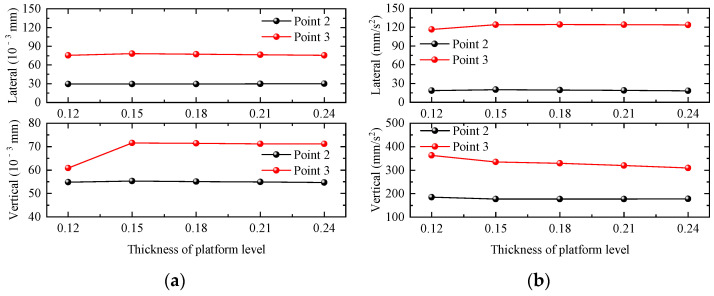
Relationship curve between station dynamic response and thickness of platform level. (**a**) Displacement; (**b**) acceleration.

In this section, five groups of platform slab thicknesses of 0.12 m, 0.15 m, 0.18 m, 0.21 m, and 0.24 m are selected to investigate the influence of platform slab thickness changes on the dynamic response of the station building structure. Taking the working condition of a single-line full-load train passing through the station at 120 km/h as an example, the maximum values of the dynamic responses of the waiting-hall slab and platform slab under different platform slab thicknesses are shown in the Figure 15. The lateral displacement and acceleration response of the waiting-hall slab and platform slab are basically unchanged with the increase in the platform floor thickness, and only the vertical displacement of the platform layer decreases with the increase in the platform floor thickness. When the thickness of the platform floor increases, the vertical displacement, lateral displacement, and lateral acceleration response of the platform floor remain basically unchanged, and only the vertical acceleration of the platform floor decreases with the increase in the thickness of the platform floor, so the responses of the waiting hall and the platform floor are less affected by the change in the thickness of the platform level.

#### 4.2.5. Section Size of Hall Layer Columns

**Figure 16:**
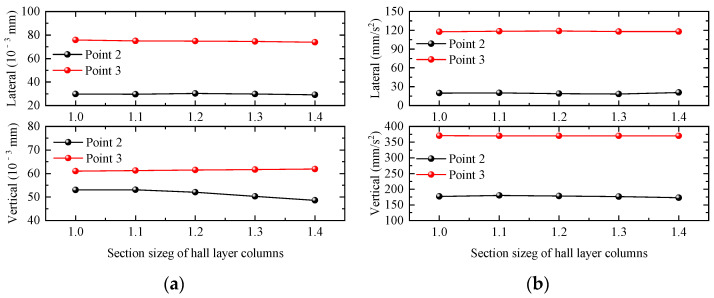
Relationship curve between station dynamic response and section size of hall layer columns. (**a**) Displacement; (**b**) acceleration.

In this section, five groups of waiting-hall frame columns of 1 m × 1 m, 1.1 m × 1.1 m, 1.2 m × 1.2 m, 1.3 m × 1.3 m, and 1.4 m × 1.4 m are selected to investigate the effects of changes in waiting-hall columns’ cross-section dimensions on the dynamic response of the station building structure. Taking the working condition of a single-line full-load train passing through the station at 120 km/h as an example, the maximum values of the dynamic responses of the waiting-hall floor slab and platform floor slab under different waiting-hall column cross-sectional dimensions are shown in Figure 16. The displacement responses of the waiting-hall floor and platform floor are basically unchanged with the increase in the waiting-hall column section size. In order to more intuitively observe the acceleration responses of the waiting-hall floor and platform floor affected by the change in the waiting-hall column section size, the acceleration dynamic response of the floor slab is plotted in the following figure. When the waiting-hall frame column cross-section size increases, it can obviously reduce the vertical displacement response of the waiting hall, while the lateral displacement, vertical displacement, and vertical acceleration of the waiting-hall and platform floor are basically unaffected.

## 5. Conclusions

In this paper, the vehicle-bridge-station spatial coupling vibration simulation model is established by using the self-developed software GSAP for the two structural station forms of integrated station-bridge and combined station-bridge elevated station program, respectively, and the simulation results are compared with the data to verify the reliability of the simulation results. The simulation results are compared with data measured on-site to verify the reliability of the simulation results in this paper. Subsequently, the relationship between the relevant parameters of the train and the dynamic response of the station is analyzed, and the relationship between some of the key design dimensions of the station structure and the dynamic response of the station in the form of “integrated station-bridge structure” is further investigated. The main conclusions are as follows:

(1) Under the same traveling condition, the vertical acceleration of the rail-bearing layer of the integrated station is smaller than that of the rail-bearing layer of the combined station, while the vertical accelerations of the platform layer and hall layer of the integrated station are opposite to each other. When the train passes through the station at low speeds of 80 km/h and 90 km/h, the vertical acceleration at the station concourse level of the two types of stations is basically the same, and the vertical acceleration at the platform level of the integrated station is obviously larger than that of the combined station;
(2) When the train passes through the station with high speed, the vertical accelerations of the platform level and station-hall level of the integrated station are larger than that of combined station under an unloaded double-line working condition, which indicates that the combined station has better dynamic performance under complicated working conditions. The combined dynamic performance of the combined station is better than that of integrated station, but there are problems such as the increased height of the whole station caused by the bridge box girder, the waterproofing problem of the track layer, the inconvenience of maintenance and replacement of the track girder support, etc. Therefore, comprehensive consideration should be given to the selection of the combined station based on various aspects;
(3) For the integrated station-bridge structure, appropriately increasing the thickness of the floor slab at the rail bearing level has the most obvious effect on the reduction in the dynamic response of the station structure, and the dynamic responses of the waiting hall and the platform are significantly reduced; followed by increasing the thickness of the floor slab at the platform level, which can significantly reduce the dynamic response of the floor slab at the platform level; and then increasing the height of the rail beams, which can significantly reduce the displacement response of the floor slab at the platform level and the floor slab at the platform hall. Increasing the thickness of the floor slab at the waiting hall and the cross-section size of the frame columns of the waiting hall has little effect on the dynamic response of the station structure. Then, increasing the heights of track beams can significantly reduce the displacement response of platform floor slabs and station-hall floor slabs, while increasing the thickness of waiting-hall floor slabs and the cross-section size of waiting-hall frame columns has little effect on the dynamic response of the station structure.

## Figures and Tables

**Table 1:** Comprehensive comparison between structures of combined and separated station-bridges.

Comparison Program	Plan A: Integrated Station-Bridge Structure	Plan B: Combined Station-Bridge Structure	Plan C: Separated Station-Bridge Structure
Load transfer relationship	The structure is cast-in-place as a whole. Train loads are dispersed through the beam-column system and then transferred to the ground floor frame columns.	The track girders are prefabricated and simply supported on the cover girders. Train loads are transferred to the cover beams and piers.	The train load is directly transferred to the bridge and is not transferred to the station.
Structural characteristics	Good structural integrity and seismic performance, seamless cast-in-place structure for track level girders and slabs	Poor structural integrity and seismic performance. There are longitudinal joints between the track girders and the main body of the station, and transverse joints between the track girders. Maintenance and overhaul of track beam supports are not easy to operate.	The bridge structure and the building structure are two completely independent systems, making the structural design relatively simple; the vibration interference from train operation on the concourse and platform levels is minimal.
Building arrangement	Flexible structural arrangement, smaller beam member sizes, less building height occupied. Relatively small column cross-section at station-hall level. Smaller lifting height of escalator.	Beam members are larger in size and take up more building height. Pier sections are generally larger. Escalator lifting heights are larger.	Beam members are larger in size and take up more building height. Pier sections are generally larger. Escalator lifting heights are larger.
Engineering investment	Relatively economical cost	Relatively higher cost	

**Table 2:** Vehicle model parameters.

Parameters	Notation
Mass of car body, mass of bogie, mass of wheelset	$M_{c},\;M_{g},\;M_{s}$
Distance between the center of gravity of the vehicle and the transverse spring.	$h_{1}$
Distance between the center of gravity of the frame and the central transverse spring	$h_{2}$
Distance between the center of gravity of the frame and the transverse spring of the axle box	$h_{3}$
Distance between the center of gravity of the wheelset and the transverse spring of the axle box	$h_{4}$
Half of the distance between the centers of gravity of the front and rear bogies.	L
Rotation inertia of car body and frame	$J_{C\psi },\;J_{g\psi }$
Half length of the wheelbase	$L_{1}$
Half the length of the train axle	$b_{0}$
Half the distance of the car body’s lateral suspension system	$b_{1}$
Half the width of the train car body	$b_{2}$
The degrees of freedom of the vehicle body’s lateral swing, floating, rolling, pitching, and yawing	$Y_{\mathrm{co}},\;Z_{\mathrm{co}},\;\theta_{\mathrm{co}},\;J_{\mathrm{co}},\;\psi_{\mathrm{co}}$
The degrees of freedom of the vehicle wheelset’s lateral swing and yawing.	$Y_{\mathrm{gh}},\;Z_{\mathrm{gh}},\;\theta_{\mathrm{gh}},\;J_{\mathrm{go}},\;\psi_{\mathrm{gh}}$
The degrees of freedom of the vehicle frame’s lateral swing and yawing	$\psi_{\mathrm{wh}},\;Y_{\mathrm{wh}}$
Stiffness of the suspension systems between the car body and the bogie	$K_{\mathrm{ux}},\;K_{\mathrm{uy}},\;K_{\mathrm{uz}}$
Stiffness of the suspension systems between the bogie and wheelset	$K_{\mathrm{dx}},\;K_{\mathrm{dy}},\;K_{\mathrm{dz}}$
Damping coefficient between the car body and the bogie	$C_{\mathrm{ux}},\;C_{\mathrm{uy}},\;C_{\mathrm{uz}}$
Damping coefficient between frame and wheelset	$C_{\mathrm{dx}},\;C_{\mathrm{dy}},\;C_{\mathrm{dz}}$

**Table 3:** Parameters of American track irregularity spectra with grade-6.

Parameters	Value	Unit
*A_v_*	0.0339	cm^2^/(rad/m)
*A_a_*	0.0339	cm^2^/(rad/m)
Ω*_s_*	0.438	rad/m
Ω*_c_*	0.8245	rad/m
Speed allowance	176	km/h

**Table 4:** Composition of different types of station models.

	Integrated Station-Bridge System	Combined Station-Bridge System
Number of nodes	5149	5573
Number of beam elements	5077	5089
Number of shell elements	4152	4152

**Table 5:** Natural vibration frequency of station model.

Order	Mode Shape	Mode Frequency (Hz)	
		Integrated Station-Bridge System	Combined Station-Bridge System
1	First-order antisymmetric transverse bending of the main structure	1.534	1.414
2	First-order longitudinal drift for beams, columns and canopies	1.542	1.430
3	Second-order transverse bends in the main structure	1.685	1.538
4	Longitudinal drift of the second order of the beam canopy	2.369	2.161
5	Third-order transverse bends in the main structure	2.829	2.513
6	Fourth-order transverse bends in the main structure	2.964	2.635
7	Column structure transverse bending	3.633	3.633
8	Canopy first-order vertical bend	3.763	3.763
9	Canopy second-order vertical bend	3.862	3.862
10	Canopy third-order vertical bend	4.044	4.044
11	Canopy third-order vertical bend	4.394	4.267

**Table 6:** Comparison of calculated and theoretical values of peak acceleration of station structures.

Measurement Point	Peak Acceleration (mm/s^2^)	
	Measured Value	Calculated Value
Point 1	42.46	32.98
Point 2	50.96	38.37
Point 3	123.86	152.57
Point 4	107.75	101.50

**Table 7:** Train calculation condition description.

Table	Train Operating Conditions	Train Formation	Vehicle Weight	Vehicle Velocity (km/h)	Track Irregularities
Type B vehicle	Single-lane passages	Four-car consist train (T + M + M + T)	Dead-load, Over-load	80, 90, 100, 110, 120	American 6-level spectrum track irregularity
Type B vehicle	Double-lane passages				

M is motor car, and T is trailer car.

**Table 8:** Maximum dynamic response of station structure under single-line condition.

Station Area	Vehicle Velocity (km/h)	Vertical Acceleration (mm/s^2^)			
		Dead-Load		Over-Load	
		Plan A	Plan B	Plan A	Plan B
Rail bearing layer	80	581.03	604.25	857.06	912.95
	90	636.99	657.74	923.03	963.14
	100	660.38	698.78	937.85	998.96
	110	666.62	683.07	946.03	984.41
	120	612.79	618.12	891.85	915.62
Hall layer	80	101.71	77.70	102.96	100.49
	90	92.94	81.87	111.48	95.98
	100	127.37	74.50	136.90	83.21
	110	175.32	99.61	206.19	126.83
	120	186.93	94.66	188.63	116.61
Platform level	80	151.66	122.42	142.76	134.02
	90	145.23	97.20	137.49	110.71
	100	146.84	90.83	167.31	116.95
	110	215.00	114.73	233.41	151.25
	120	237.26	138.27	226.36	166.55

**Table 9:** Maximum dynamic response of station structure under double-line condition.

Station Area	Vehicle Velocity (km/h)	Vertical Acceleration (mm/s^2^)			
		Dead-Load		Over-Load	
		Plan A	Plan B	Plan A	Plan B
Rail bearing layer	80	584.37	601.73	860.85	909.12
	90	637.48	660.08	924.81	965.94
	100	661.37	699.47	939.15	999.65
	110	665.69	684.43	945.53	985.65
	120	613.19	618.57	892.55	916.40
Hall layer	80	113.59	76.65	106.46	99.32
	90	92.62	89.76	104.37	98.70
	100	131.08	77.88	143.00	97.40
	110	192.26	127.60	223.70	149.57
	120	214.01	149.92	233.03	193.67
Platform level	80	170.05	120.78	169.31	131.25
	90	174.51	117.55	168.83	132.82
	100	178.47	101.56	199.95	140.24
	110	258.18	149.72	285.04	172.53
	120	314.16	178.43	319.12	192.19

## Data Availability

All data included in this study are available upon request by contact with the corresponding author.
